# Fabrication and Anti-Oxidation Ability of SiC-SiO_2_ Coated Carbon Fibers Using Sol-Gel Method

**DOI:** 10.3390/ma11030350

**Published:** 2018-02-27

**Authors:** Guangyuan Yang, Zhixiong Huang, Xu Wang, Bo Wang

**Affiliations:** Department of Materials Science and Engineering, Wuhan University of Technology, Wuhan 430070, China; huangzhixiong@whut.edu.cn (Z.H.); wangxu123@whut.edu.cn (X.W.); whbowang189@gmail.com (B.W.)

**Keywords:** carbon fiber, oxidation resistance, coating, characterization

## Abstract

The paper proposed a method to improve the anti-oxidation performance of carbon fibers (CF) at high temperature environment by coating silicon dioxide (SiO_2_) and silicon carbide (SiC). The modified sol-gel method had been used to ensure the proper interface between fibers and coating. We used polydimethylsiloxane and ethyl orthosilicate to make stable emulsion to uniformly disperse SiC nanoparticles. The modified SiO_2_/SiC coating had been coated on CF successfully. Compared with the untreated CF, the coated fibers started to be oxidized around 900 °C and the residual weight was 57% at 1400 °C. The oxidation mechanism had been discussed. The structure of SiC/SiO_2_ coated CF had been characterized by scanning electron microscope and X-ray diffraction analysis. Thermal gravimetric analysis was used to test the anti-oxidation ability of CF with different coatings.

## 1. Introduction

Since the 1960s, carbon fibers (CF) have been widely used in various fields due to its high specific modulus, corrosion resistance and electrical conductivity as well as other unique properties. Instead of using CF alone, people tend to combine it with different matrices to obtain composites that own excellent strength, low thermal expansion coeffient, and lightweight [1,2,3]. In the aerospace area, some parts of planes, spaceships, and some parts of rocket need to resist extremely high temperature. For example, the rocket fairing is used to protect the rocket and the satellite from damages caused by heat out of its friction in the atmosphere. Traditional ultra-high temperature protection materials contain refractory metal, ceramic composites, et cetera [4]. They were either too heavy, or the mechanical properties were not very good. CF reinforced composites have great potential to replace them because of lightweight, high-strength and flexibility. The rocket fairing of Titan rocket (USA) was made from carbon fibers, epoxy resin, and aluminum honeycomb. However, CF has poor oxidation resistance in the air environment at high temperature. It is important to improve the anti-oxidation ability of CF to extent its application to make thermal protection parts with excellent mechanical properties. 

Shielding is an effective method to increase the oxidation resistance ability of CF. It can prevent CF substrate from contacting the oxygen directly. The type of coating can be various, such as metal coating (Zn, Ni, Cu, Ti, Zr, Ag); oxides coating (SiO_2_, Fe_3_O_4_, Al_2_O_3_, TiO_2_), nitrides coating (BN, TiN), carbides coating (SiC, TiC, TaC, ZrC, B_4_C), and composite coating (Al_2_O_3_/Y_2_O_3_, ZrC–ZrB_2_–SiC, SiBNC) [5,6,7,8,9,10,11,12,13,14,15,16]. Among them, ceramic coatings are the most promising coatings due to the fact that they are much lighter and have better wettability, which can reduce interface diffusion and reaction. 

The common ways to fabricate ceramic coatings are vapor deposition, precursor infiltration, and pyrolysis (PIP), and sol-gel method [17,18,19]. The chemical or physical vapor deposition can produce uniform and dense coatings. Moreover, plasma treatment has been used in vapor deposition, which can deposit homogeneous, well-adhesive coatings at lower temperature on different substrates [20,21]. To improve the surface roughness, structure, and mechanical properties of CF, hydrogen and oxygen plasma treatments are applied. The density of functional groups and changes in the carbon bonding contributed to the enhancement of the adhesion to PEI matrix [22]. Both the morphology and the structure of coatings are controllable. But, the low deposition rate and the uneconomical cost make vapor deposition not always practical [23]. In PIP, polycarbosilane (PCS) and polysilazane (PSZ) are often used as precursor to make SiC coating. However, it is hard for the polymer precursor to synthesize because of the complex process [24,25]. Sol-gel method is a much more practical and feasible way to fabricate three-dimensional (3D) composite coating. Besides, the cost of Sol-gel method is quite economical, and the coating has a low densification temperature (<1000 °C) and low shrinkage, which can reduce drying stress between coatings and matrix. Some reseachers [26,27,28,29] mentioned that the tetraethylorthosilicate (TEOS), vinyltriethoxysilane (VTES) and ethyl alcohol (EtOH) could be used as raw materials to fabricate SiO_2_ coating by sol-gel method. However, the best decomposition temperature they have got is around 800 °C. When the temperature is over 600 °C, the weight loss is more than 50%. The anti-oxidation performance of silicon materials coated CF still has great potential to improve. 

To lower the formation temperature and to improve the anti-oxidation performance of CF, modified sol-gel method has been proposed in this paper. The best ratio of TEOS/water has been discussed. The mechanism of oxidation process and the properties of coated CF at different temperature environment have been investigated.

## 2. Experiment

### 2.1. Preparation of Modified SiC/SiO_2_ Sol and Emulstion

To remove the protective polymer layer of CF (Wuxi Weppom Composite Materials Company, Wuxi, China), the fibers were heated at 400 °C for 1 h under the nitrogen atmosphere. Then, put the CF into 75 wt % nitric acid (Shanghai Aladdin Reagent Factory, Shanghai, China) with ultrasonic treatment for 1 h at 60 °C. 

In order to explore the optimum proportion of sol, four different samples were prepared under different ratio of raw materials. The samples had been marked as samples 1–4 with the ratio of ethanol (Sinopharm Chemical Reagent Company, Shanghai, China), distilled water and TEOS (Shanghai Aladdin Reagent Factory) being 5:1:1, 5:1:3, 5:1:5, 5:1:7, respectively. Hydrochloric acid had been used to adjust pH to 3. After stirring the solution for 2 h, the sol had been stood for 10 h at 60 °C to obtain transparent SiO_2_ sol. 

To disperse SiC nanoparticles (50nm, Shanghai Aladdin Reagent Factory) uniformly, homogenizer (15,000 rpm) was used to prepare SiO_2_ emulsion. The polydimethylsiloxane (Shanghai Aladdin Reagent Factory, ~10 mPa.s, neat, s104472), SiO_2_ sol and distilled water were mixed with the ratio 1:1:1. 5 wt % tween 80 (T104866, Shanghai Aladdin Reagent Factory) had been added into the mixture as emulsifier. When the SiO_2_ emulsion became stable, SiC nanoparticles were dispersed into the emulsion (24 g/200 mL) uniformly. 

### 2.2. Coating Process

CF was immersed into the SiC/SiO_2_ emulsion separately. After 30 min ultrasonic treatment, the fiber was pulled out from the sol (10 mm/min) and heated to 600 °C (5 °C/min) in argon flow (40 mL/min). After 30 min preservation, the temperature increased to 1200 °C with a rate of 4 °C/min. Before naturally cooling down to the room temperature, the sample was preserved at 1200 °C for 120 min. Then, repeat the above steps 3 times to increase the thickness of coating.

### 2.3. Oxidation

In order to explore the mechanism of oxidation process, SiC/SiO_2_ coated CF samples were treated at 800 °C, 1000 °C and 1400 °C for 15 min in the air environment. To evaluate the influence of thermal shock, the samples were put back to room temperature and cooled down naturally.

### 2.4. Characterization

The surface morphology of coated CF and oxidized samples were characterized by a JSM-7800FPRIME Scanning Electron Microscope (SEM) (JEOL, Tokyo, Japan). To analyze the crystal structure of unoxidized coating and 1400 °C treated coating, the X-Ray diffraction (XRD) analysis (D8 Advance X-ray diffract meter, Bruker Corporation, Ettlingen, Germany) was used. The scanning was conducted from 2θ angle of 5°–80° at a scan rate of 5°/min. The NETZSCH STA 449 F3 typed thermal analyzer (NETZSCH, Selb, Germany) was applied to Thermal Gravimetric (TG) analysis. It can test the anti-oxidation performance of each sample (both uncoated CF and SiC/SiO_2_ coated CF) under the air atmosphere with a constant heating rate of 5 °C/min from 25 °C to 1300 °C. 

To test the mechanical property of CF, 10 samples were prepared by the method in this patent [30]. First, CF had been fixed at two sides of a frame. Then, the frame was immersed into 40 wt % epoxy resin for 5 min. After drying at 110–130 °C, the CF was tested through the Instron testing machine (model-5866, Instron Pty Ltd, Norwood, MA, USA). The testing process was followed by the ISO standard [31]. The tensile strength was calculated by the equation:

(1) $$\sigma =\frac{P-\rho }{t}\times 10^{6}$$

σ—tensile strength (MPa); P—failure load (N); ρ—the density of CF (kg/m^3^); t—linear density of CF (kg/m).

The Young’s modulus was calculated by the equation:

(2) $$E=\frac{\Delta P-\rho }{t}\times \frac{L}{\Delta L}\times 10^{-9}$$

E—Young’s modulus (GPa); ΔP—The change of load value (N); ρ—the density of CF (kg/m^3^); t—linear density of CF (kg/m); L—the length of the sample (mm); and, ΔL—The change of length of the sample (mm).

The fracture elongation was calculated by the equation:

(3) $$\varepsilon =\frac{\Delta L_{b}}{L}\times 100\%$$

Ɛ—the fracture of elongation; L—the length of the sample (mm); ΔL_b_—elongation at break.

## 3. Result and Discussion

### 3.1. Microstructure Analysis of Coatings

**Figure 1:**
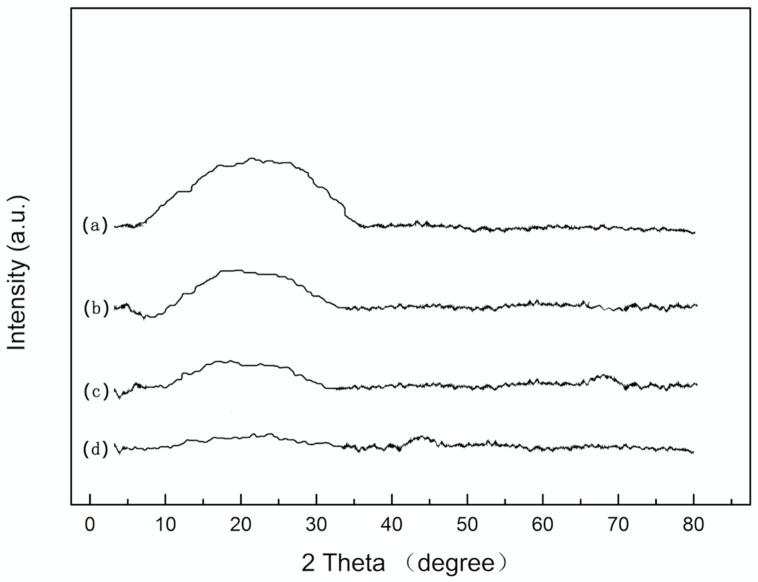
X-ray diffraction (XRD) analysis of silicon dioxide (SiO_2_) coating with different Water/TEOS ratio (**a**) 1/3; (**b**) 1/1; (**c**) 1/5; (**d**) 1/7.

Table 1 presents the coating properties of different water/TEOS ratio. Figure 1 shows that the XRD diffraction patters of coating between 15° and 30° is wide. It can be deduced that the main component of the coating is amorphous SiO_2_. The curve of line (a) is wider and more obvious than other three lines. It means that the formation rate is the fastest with the ratio 1/3. When combined with the appearance and performance results that were shown in Table 1, it can certify that best water/TEOS ratio to fabricate SiO_2_ coating is 1/3. The main reactions of the sol-gel system were as follows.

Hydrolytic reaction:

(4) $$\mathrm{Si}{\left(\mathrm{OR}\right)}_{4}+4H_{2}O\to \mathrm{Si}{\left(\mathrm{OH}\right)}_{4}4\mathrm{ROH}$$

Condensation reaction:

(5) $$2\mathrm{Si}{\left(\mathrm{OH}\right)}_{4}\to \mathrm{Si}{\left(\mathrm{OH}\right)}_{3}–O–\mathrm{Si}{\left(\mathrm{OH}\right)}_{3}+H_{2}O$$

Polymerization: 
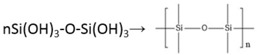
(6)

Among them, R stands for –C_2_H_5_. The products of the reactions are colloidal particles with different size and structure. A three-dimentional network of xerogel can be produced after dehydration. The property of xerogel is related to the temperature, solvent, pH and the ration of water and TEOS. TEOS cannot dissolve in water, but it can dissolve in EtOH and react with water in it. When the water is much more than the TEOS, it will take a long time to form sol. Instead, when the water is much less than the TEOS, sol can be form very quickly (less than 40 min). However, the stability of such sol is poor, and stratification could happen in the few hours. So, the SiO_2_ sol was prepared with the best water/TEOS ratio.

**Figure 2:**
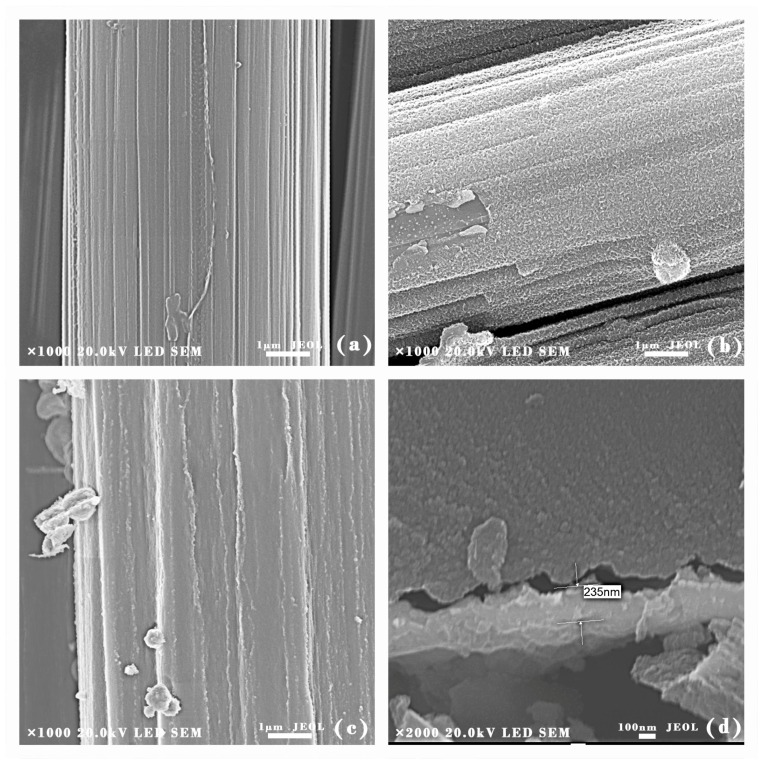
Scanning Electron Microscope (SEM) images of (**a**) uncoated carbon fibers (CF); (**b**) SiO_2_ coated CF; (**c**) SiC/SiO_2_ coated CF; and, (**d**) cross-section of coated CF.

**Figure 3:**
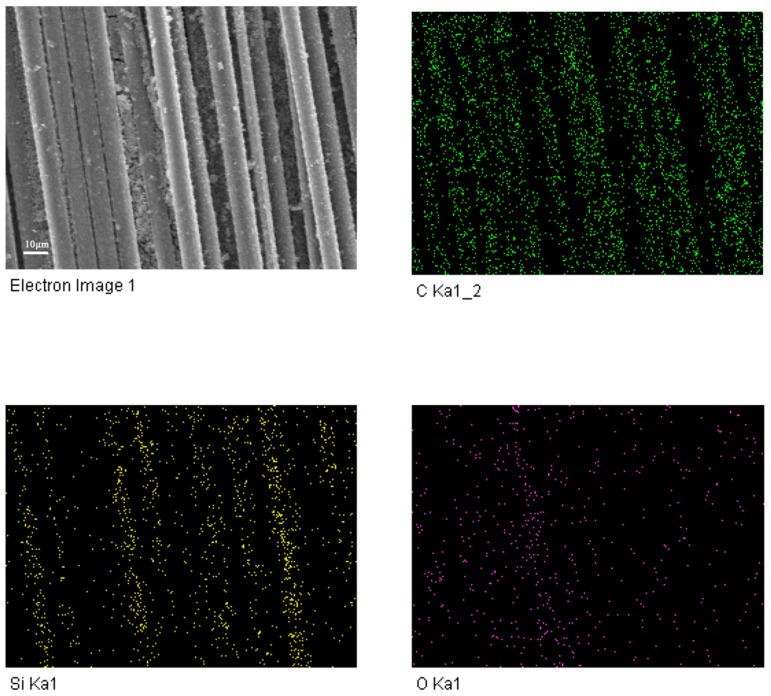
EDS analysis of SiC/SiO_2_ coated CF. Scale bar represents 10 µm.

To analyze the surface morphology of the coatings, SEM experiments were conducted on uncoated CF and SiO_2_ coated CF. Figure 2a shows that the grooves were formed at the surface of uncoated CF. Because, after removing the protective layer of CF, concentrated nitric acid could increase the surface roughness and specific area of CF, enhancing the interface effect between SiO_2_ coating and CF. The generation of carboxylic acids, hydroxyl groups, and lactones functionalities contributed to the increasing of adhesion strength [32]. Figure 2b presents the surface morphology of SiO_2_ coated CF. Since the SiO_2_ coating had covered the grooves; the surface of CF was uniform and smooth. However, some aggregations could be seen at the surface of the coating, since the emulsion particles were aggregated and may not spread uniformly before the heating process. SiC/SiO_2_ coated CF are shown in Figure 1c. The SiC nanoparticles had been coated onto CF successfully. The surface of SiC/SiO_2_ coated CF was rough and laminar. There were some small bulges that were spreading along the CF as the SiC nanoparticles had been embedded into SiO_2_ coating. Figure 2d was the cross-section morphology of coated CF. The coating was compact and uniform. Displacement can be found at the interface between coating and CF. The coating had been moved to the left side of the picture because of the external force when the CF was cut off. The protrusion and concave can fit together on the whole. Therefore, it can be deduced that the coating was originally attached on CF closely. It can be found that the coating was compact and uniform. The whole coating is about 0.23 μm in thickness, and there was little aggregation at the surface of the coating. Energy Dispersive Spectroscopy (EDS) analysis had been used to further analyze the elements distribution of composite coating. Figure 3 shows that Si, O, and C were dispersed evenly on the surface of the fiber. Because of the existence of SiC nanoparticles, O element was not as concentrated as Si and C elements.

**Figure 4:**
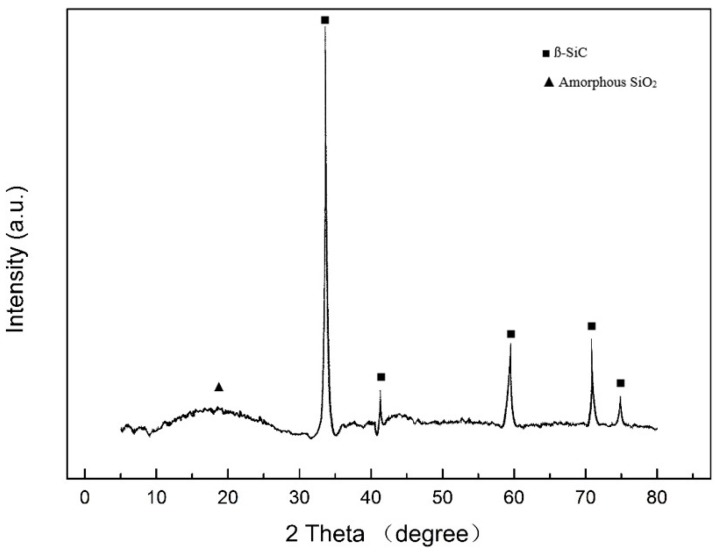
XRD images of SiC/SiO_2_ coated CF.

Figure 4 shows the band diffraction peaks of SiC/SiO_2_ coated CF. Around 2θ = 22°, the peak was broad and wide, which reflects the existence of amorphous SiO_2_. The formation temperature is not high enough to make SiO_2_ emusltion to transit to crystal. At 35°, 41°, 60°, and 71°, the diffraction peaks were corresponded to the ß-SiC. On the one hand, it proves that SiC nanoparticles had embedded into the coating successfully; on the other hand, it can extrapolate that the nanoparticles and the coating only had physical bonding instead of chemical reactions. 

### 3.2. Mechanical Property of Coated CF

The mechanical property was tested following the method described in reference [30,31]. The results were shown in the Table 2. It could be observed that there are little differences in the mechanical property of coated CF and uncoated CF as coating only affected the roughness of CF’s surface, because the coating only affected the roughness of CF’s surface. From the XRD analysis (Figure 4), it proves that the interfacial bonding between the coating and CF is physical bonding. The strength of ceramic is higher than CF. Besides, the ceramic coating may cover some surface defects and limit their influence on the crack initiation [33]. So, before oxidation, the coated CF owned higher strength than the uncoated CF. When the temperature was at 800 °C, the mechanical property of coated CF only decreased 15%. However, when the temperature was at 1000 °C, the mechanical property of coated CF decreased rapidly, especially the modulus. Since the coating was partly damaged and the stiffness of coated CF had been reduced, it became much easier for the fiber to be deformed by external force. While the temperature was over 1400 °C, the fibers became soft and brittle. CF had been seriously damaged and the cracks of the coating could be seen directly at the surface of CF. The mechanical property of coated fibers slumped quickly. Based on the above results, the mechanical property of SiC/SiO_2_ coated CF will decrease after oxidation. However, when the temperature was lower than 1400 °C, the strength and the modulus of coated CF were in the acceptable range so the CF could stay in the right shape.

### 3.3. Anti-Oxidation Performance

**Figure 5:**
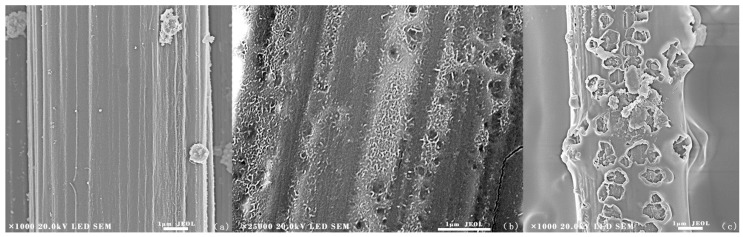
SEM micrograph of the surface of (**a**) SiC/SiO_2_ coated CF(800 °C,15 min); (**b**) SiC/SiO_2_ coated CF(1000 °C,15 min); and, (**c**) SiC/SiO_2_ coated CF(1400 °C,15 min).

**Figure 6:**
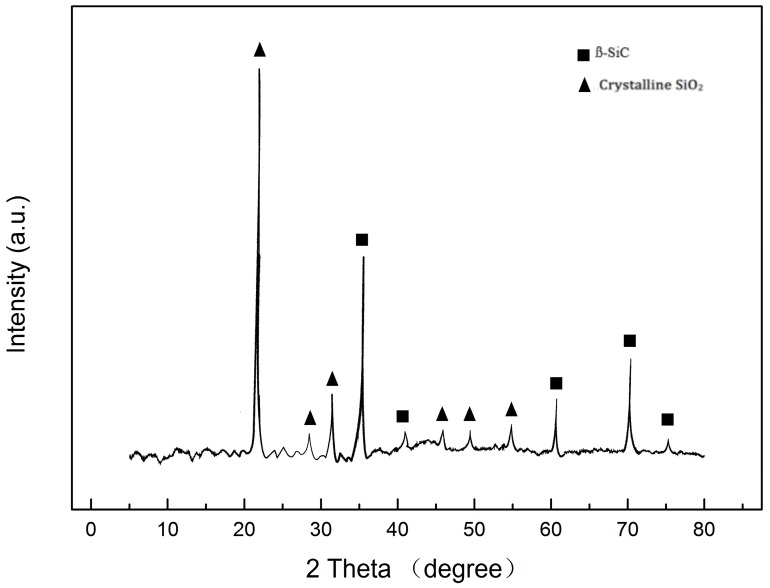
XRD analysis of SiC/SiO_2_ coated CF with 1400 °C treated for 15 min.

SiC/SiO_2_ coated CF were treated at 800 °C, 1000 °C and 1400 °C in the air environment for 15 min. Figure 5a shows the SEM image of the SiC/SiO_2_ coated CF heated at 800 °C for 15 min. The fibers stayed in the right shape and there were no cracks or pores at the surface, which means that the SiC/SiO_2_ coating had protected fibers well. From Figure 5b, the surface of 1000 °C treated SiO_2_ coated CF was more coarse and little cavities could be found due to the gas that had broken through the coating and some SiC nanoparticles had been peeled off from the coating. However, these cavities did not penetrate the coating, so the oxygen could not react with CF directly. The coated CF had acceptable mechanical property for such an environment, indicating that 1000 °C can be the proper temperature in the real application. Figure 5c presents the surface morphology of coated CF after 15 min 1400 °C treatment. It can be found that pores and cracks were distributed on the coating. The diameter of pores could be over 1 µm, which indicated that the coating had been seriously damaged. It can be deduced that the gas was formed inside the coating due to the edge of the pores was convex. By penetrating into the coating, oxygen could react with CF, leading the rapid oxidation of the fibers. Combine with XRD results (Figure 6). It can be concluded that although higher temperature had contributed to the formation of crystalline SiO_2_ at the surface of the coating, the inner oxidation still destroyed the anti-oxidation ability of the coating due to the gas formation.

**Figure 7:**
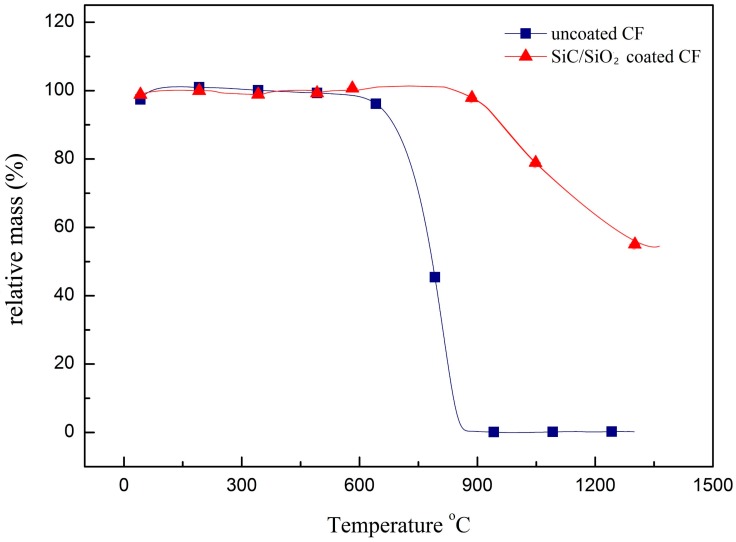
Thermal Gravimetric (TG) curves of uncoated CF and SiC/SiO_2_ coated CF.

Figure 7 is the TG analysis of CF with different coatings. The results show that SiC/SiO_2_ coating can effectively improve the anti-oxidation ability of CF in the air environment. Uncoated CF began to lose weight when the temperature was over 400 °C. The TG curve of uncoated CF started to decrease rapidly and decomposed completely around 850 °C. The SiC/SiO_2_ coated CF started to lose weight at 900 °C. Around 1400 °C, the residual weight of SiC/SiO_2_ coated CF was more than 57%. Moreover, the mass loss of coated CF was smaller, which means the oxidation rate of coated CF is slower than uncoated CF. When compared with the temperature of other silicide coatings that were fabricated with the traditional sol-gel method, the decomposition temperature of SiC/SiO_2_ coated CF had been improved significantly.

### 3.4. Oxidation Mechanism of Coated CF

**Figure 8:**
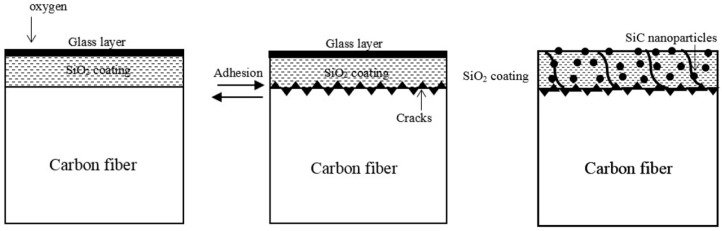
Oxidation mechanism of coated CF.

Figure 8 is the sketch of the cross-section of CF. It shows the oxidation mechanism of coated CF. From other report [34], the coating of thermal protection system should have a high melting point, low density, thermal shock resistance, and low thermal conductivity. Besides, the thickness of coating should be considered based on the purpose of the CF. To ensure the acceptable mechanical property of CF, the coating cannot be too thick. 

When it comes to the samples that we prepared, both SiO_2_ and SiC had relative high melting points and SiC had similar coefficient of thermal expansion that makes them perfect to be used as thermal protection materials. From the mechanical test, it can be deduced that the thickness of SiC/SiO_2_ coating was appropriate. While the temperature increased, the silicon dioxide layer will be formed at the surface of the coating. The glass layer can protect the inner coating by preventing oxygen from penetration. When the temperature was lower than 1000 °C, the CF stayed in the right shape. Only small pores would be formed at the surface of the coating due to part of SiC nanoparticles reacting with oxygen to form CO_2_ and CO. However, once the temperature was over 1000 °C, the accumulated gases could lead cracks and pores in the coating. When oxygen contacted with CF, a large amount of gases destroyed the integrality of the coating. Besides, the coefficients of thermal expansion were different between the coating and CF substrate. With the temperature increased, thermally grown oxides could be formed at the interface of the coating and CF substrate, which leads to higher interface stress and a decrease in the strength of interfacial bonding. Some part of the coating might be peeled off from the CF. What’s more, all of the samples were put back to the room temperature as soon as the oxidation finished. The planar cracks could be formed by the thermal shock. Although SiC nanoparticles have great oxidation resistance potential, while the temperature is high enough, the activation energy of the particles can cause cracks along the interface. Once the oxygen came in the inner coating, SiC nanoparticles can form CO and CO_2_, which would damage the coating and accelerate the oxidation speed of CF.

## 4. Conclusions

This study proposed a modified sol-gel method to fabricate SiC/SiO_2_ coating at the surface of CF. The coating can improve the oxidation resistance ability of CF at high temperature environment. The best TEOS/water ratio to make SiO_2_ sol had been determined. Attributed to the SiC/SiO_2_ coating, the anti-oxidation performance of CF had been increased. The residual weight of coated CF was more than 57% when the temperature was 1400 °C. The mechanical property of SiC/SiO_2_ coated CF only had little differences between uncoated CF when the temperature was lower than 1000 °C. In the future, to reach better anti-oxidation ability of coated CF, a new method should be developed to increase the interface strength between coating and the CF and lower the formation temperature of coating. 

## Figures and Tables

**Table 1:** Result of samples with different water/TEOS ratio.

Sample Number	Water/TEOS	SiO_2_ Coating
Sample 1	1/1	The thickest coating with no cracks. The surface is not uniform.
Sample 2	1/3	Thinner than Sample 1, and the surface is uniform.
Sample 3	1/5	The surface is not uniform, and cracks appear.
Sample 4	1/7	It cannot form SiO_2_ coating.

**Table 2:** Mechanical property of different CF.

Sample	Strength MPa	Young’s Modulus GPa	Fracture Elongation %	Standard Deviation of Modulus
Uncoated CF	2745	223	1.75	5.98
SiO_2_ coated CF	2980	207	1.40	4.24
SiC/SiO_2_ coated CF	3098	210	1.43	5.12
800 °C treated SiO_2_ coated CF	2455	132	1.22	3.35
1000 °C treated SiO_2_ coated CF	1832	101	1.16	4.31
1400 °C treated SiO_2_ coated CF	1102	67	0.65	4.89
